# Epetraborole Is Active against Mycobacterium abscessus

**DOI:** 10.1128/AAC.01156-21

**Published:** 2021-09-17

**Authors:** Uday S. Ganapathy, Martin Gengenbacher, Thomas Dick

**Affiliations:** a Center for Discovery and Innovation, Hackensack Meridian Health, Nutley, New Jersey, USA; b Department of Medical Sciences, Hackensack Meridian School of Medicine, Nutley, New Jersey, USA; c Department of Microbiology and Immunology, Georgetown University, Washington, DC, USA

**Keywords:** epetraborole, *Mycobacterium abscessus*, NTM, nontuberculous mycobacteria, benzoxaborole

## Abstract

Benzoxaboroles are a new class of leucyl-tRNA synthetase inhibitors. We recently reported that the antitubercular 4-halogenated benzoxaboroles are active against Mycobacterium abscessus. Here, we find that the nonhalogenated benzoxaborole epetraborole, a clinical candidate developed for Gram-negative infections, is also active against M. abscessus *in vitro* and in a mouse model of infection. This expands the repertoire of advanced lead compounds for the discovery of a benzoxaborole-based candidate to treat M. abscessus lung disease.

## INTRODUCTION

Mycobacterium abscessus lung disease is notoriously difficult to treat due to the bacterium’s high intrinsic drug resistance (1, 2). In addition to resistance to all first-line tuberculosis (TB) drugs, M. abscessus displays resistance to macrolides (3, 4), threatening the current macrolide-based treatment regimens (2, 5). Therefore, new antibiotics with novel targets and mechanisms of action are needed to treat this disease (6).

**FIG 1:**
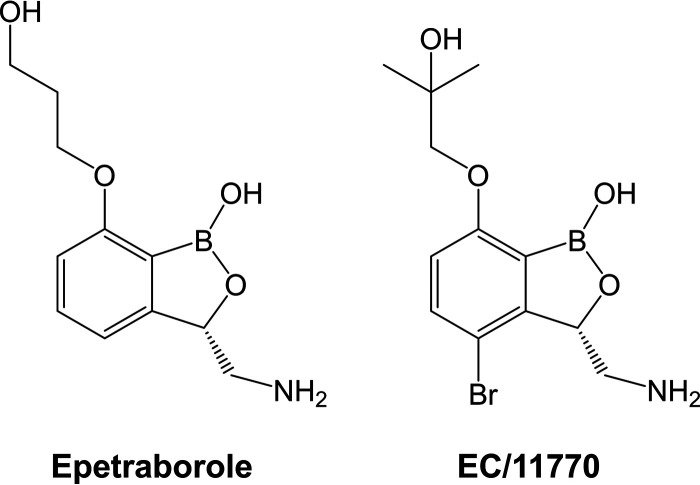
Structures of epetraborole and EC/11770.

Benzoxaboroles are a class of boron-heterocyclic antimicrobials that target leucyl-tRNA synthetase (LeuRS) (7). Acting through the oxaborole tRNA-trapping (OBORT) mechanism (8), these compounds form adducts with uncharged tRNA^Leu^ molecules that subsequently bind to the LeuRS editing domain, blocking protein synthesis. Following the discovery of tavaborole (7, 8), a benzoxaborole with antifungal activity, this compound class was optimized for antibacterial activity. Addition of a 3-aminomethyl group to the benzoxaborole core improved interactions with the editing domain of Escherichia coli LeuRS, while a 7-*O*-propanol substituent added a novel interaction with the phosphate backbone of tRNA^Leu^ (9). Combining these modifications yielded epetraborole (Fig. 1), a clinical candidate with potent activity against a broad range of Gram-negative bacteria (9, 10). The subsequent addition of a 4-halogen group (particularly Cl or Br) improved antituberculosis activity (11–13).

Recently, we reported that the antituberculosis 4-halogen benzoxaborole EC/11770 (Fig. 1) is active against M. abscessus *in vitro* and *in vivo* in a mouse infection model (14). Here, we asked whether the anti-Gram-negative, nonhalogenated benzoxaborole epetraborole (Fig. 1) is active against M. abscessus. We first measured the MIC of this compound against our screening strain M. abscessus subsp. *abscessus* Bamboo (15) in Middlebrook 7H9 medium using 96-well plates, as previously described (14). Surprisingly, epetraborole showed activity similar to that of the antitubercular EC/11770 (Table 1). Epetraborole retained activity against culture collection reference strains for each of the three subspecies of the M. abscessus complex and a panel of M. abscessus clinical isolates (16, 17) (Table 1). Taken together, the anti-Gram-negative, nonhalogenated benzoxaborole epetraborole was active against the M. abscessus complex *in vitro*.

**TABLE 1:** Activity of epetraborole against members of the M. abscessus complex

Strain	Strain type	MIC (μM)^*a*^		
		CLR	EC/11770^*b*^	EPB
M. abscessus Bamboo	Clinical isolate, screening strain	0.30	1.2	0.28
M. abscessus subsp. *abscessus* ATCC 19977	Culture collection reference strain	0.90	0.70	0.33
M. abscessus subsp. *massiliense* CCUG 48898^T^	Culture collection reference strain	0.22	0.71	0.32
M. abscessus subsp. *bolletii* CCUG 50184^T^	Culture collection reference strain	1.3	1.3	0.49
M. abscessus subsp. *abscessus* M9	Clinical isolate	1.4	0.49	0.42
M. abscessus subsp. *abscessus* M199	Clinical isolate	3.3	0.93	0.56
M. abscessus subsp. *abscessus* M337	Clinical isolate	1.6	0.50	0.44
M. abscessus subsp. *abscessus* M404	Clinical isolate	0.2	0.52	0.3
M. abscessus subsp. *abscessus* M422	Clinical isolate	0.68	0.33	0.34
M. abscessus subsp. *bolletii* M232	Clinical isolate	1.6	0.67	0.37
M. abscessus subsp. *bolletii* M506	Clinical isolate	0.28	0.48	0.28
M. abscessus subsp. *massiliense* M111	Clinical isolate	0.25	0.95	0.44
M. abscessus subsp. *abscessus* K21	Clinical isolate, infection model	0.78	0.60	0.40

a MIC values are the means from two independent experiments. CLR, clarithromycin; EPB, epetraborole.

b EC/11770 MIC values are from published literature (14) and are included for comparison.

To confirm that epetraborole indeed exerts its antimycobacterial activity by targeting M. abscessus LeuRS, we selected for epetraborole-resistant M. abscessus mutants (Table 2). Adapting our previously described method (14), M. abscessus ATCC 19977 culture was plated on Middlebrook 7H10 agar containing 16.5 μM epetraborole, the lowest concentration suppressing the emergence of wild-type colonies. After 5 days of incubation, apparent resistant colonies were confirmed by restreaking on epetraborole-containing agar. Based on two independent selections, we calculated the frequency of resistance to epetraborole to be 5.4 × 10^−8^/CFU. This frequency of resistance was on the lower end of a range determined for epetraborole in several Gram-negative bacterial species (3.8 × 10^−8^/CFU to 8.1 × 10^−7^/CFU) (9) and was comparable to what we reported for EC/11770 in M. abscessus (3.9 × 10^−8^/CFU) (14). MIC profiling of nine epetraborole-resistant mutants (RM1 to −9) showed high-level resistance to epetraborole (Table 2). Sequencing of *leuS* (*MAB_4923c*) showed that RM1 to −9 all had missense mutations in the LeuRS editing domain (residues V292 to K502) (Table 2). These results suggest that epetraborole retains LeuRS as its target to exert its anti-M. abscessus activity (8, 9).

**TABLE 2:** Characterization of M. abscessus epetraborole-resistant mutants

Strain	Batch	MIC (μM)^*a*^		LeuS mutation	Other bacteria with LeuS mutation (reference)^*b*^
		CLR	EPB		
M. abscessus ATCC 19977		1.3	0.48	None	
RM1	1	1.5	>100	LeuS G393V	None
RM2	1	2.7	>100	LeuS T322I	E. coli, Proteus mirabilis (18)
RM3	1	1.3	>100	LeuS T323P	None
RM4	2	1.5	>100	LeuS S303L	M. tuberculosis (11)
RM5	2	1.8	>100	LeuS S303L	M. tuberculosis (11)
RM6	2	1.6	>100	LeuS S303L	M. tuberculosis (11)
RM7	2	1.4	>100	LeuS Y421D	M. tuberculosis (Y421C) (11)
RM8	2	0.9	>100	LeuS T322I	E. coli, P. mirabilis (18)
RM9	2	2.2	>100	LeuS F321V	None

a MIC values are the means from two independent experiments. CLR, clarithromycin; EPB, epetraborole.

b Corresponding benzoxaborole resistance-conferring LeuS mutations reported for other bacteria.

Development of epetraborole for the treatment of complicated urinary tract infections caused by Gram-negative bacteria was discontinued after rapid emergence of drug resistance in a phase II clinical trial (18). Determination of spontaneous resistance frequencies for epetraborole in the current study, and for EC/11770 previously (14), suggest low propensity for the development of resistance against benzoxaboroles in M. abscessus. However, it is to note that we needed to carry out selection of resistant mutants on agar containing high concentrations of the drugs (50 to 100× broth MIC), as lower concentrations did not suppress outgrowth of wild-type bacteria. Thus, it cannot be excluded that the spontaneous resistance frequency of M. abscessus against the benzoxaboroles would be higher than the observed 4 × 10^−8^ to 5 × 10^−8^/CFU when lower drug concentrations could be used. Such resistant strains, presumably displaying low level resistance, would have been missed in our selection experiments. In any case, given the use of multidrug chemotherapy in M. abscessus treatment (2, 5), the risk of benzoxaborole resistance emerging in this bacterium would be reduced significantly.

**FIG 2:**
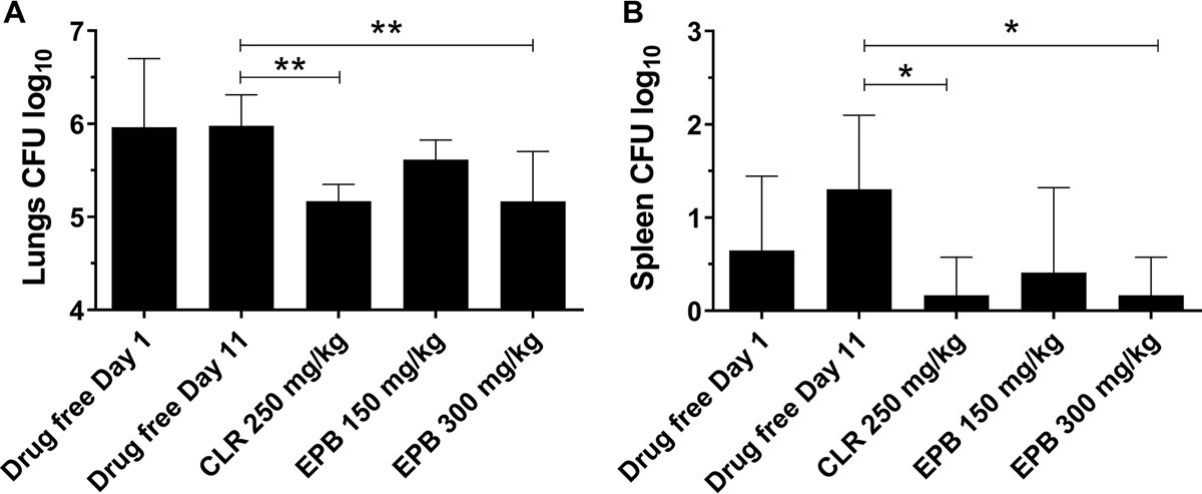
Epetraborole is active against M. abscessus *in vivo*. Lung CFU (A) and spleen CFU (B) from NOD SCID mice 1 day after intranasal infection with *Mab* (drug-free day 1) and following daily oral administration of drug-free vehicle, clarithromycin (CLR), or epetraborole (EPB) for 10 days (day 11). Data represent the means plus standard deviations from six mice per treatment group. Statistical significance of the results was analyzed by one-way analysis of variance (ANOVA) multiple-comparison and Tukey’s posttests. *, *P* < 0.05; **, *P* < 0.01.

To determine whether epetraborole is active against M. abscessus *in vivo*, we evaluated the efficacy of this compound in a previously established murine model of M. abscessus infection (17). All experiments involving live animals were approved by the Institutional Animal Care and Use Committee of the Center for Discovery and Innovation, Hackensack Meridian Health. NOD SCID mice were infected intranasally with M. abscessus K21. At day 1 postinfection, the lung bacterial burden of the mice reached ∼10^6^ CFU (Fig. 2A). Beginning on day 1, clarithromycin (formulated in 0.5% carboxymethyl cellulose-0.5% Tween 80-sterile water), epetraborole (formulated in sterile phosphate-buffered saline [PBS]), or vehicle (sterile PBS) was administered by oral gavage once per day for 10 days. Based on a previous efficacy study using a Pseudomonas aeruginosa mouse infection model (9), epetraborole was administered at 150 and 300 mg/kg body weight. The lung bacterial burden remained unchanged in mice that received the drug-free vehicle control (Fig. 2A, day 11). Mice that received epetraborole at 300 mg/kg showed a statistically significant 1-log reduction in lung CFU that was comparable to that after treatment with clarithromycin at 250 mg/kg (Fig. 2A). A similar pattern of CFU reduction was observed in the spleen (Fig. 2B). Thus, epetraborole was active against M. abscessus *in vivo*. It is interesting to note that epetraborole, despite having similar *in vitro* activity as the previously characterized benzoxaborole EC/11770 (Table 1) (14), required with 300 mg/kg a 30-fold higher dosing to achieve a similar (∼10-fold) reduction in bacterial lung burden. The basis for this difference remains to be determined but may be due to differences in the pharmacokinetic properties of the two compounds, including oral bioavailability (9, 14).

In conclusion, we show that epetraborole, an advanced nonhalogenated 3-aminomethyl benzoxaborole developed for Gram-negative infections, is also active against M. abscessus *in vitro* and in a mouse model of infection. This agrees with a recent publication that identified epetraborole in a screen of the MMV pandemic response box for anti-M. abscessus activity and reported this compound’s efficacy against M. abscessus in a zebrafish infection model (19). Our findings reaffirm leucyl-tRNA synthetase as an attractive target against M. abscessus and expand the repertoire of advanced lead compounds for the discovery of a benzoxaborole-based candidate for the treatment of M. abscessus lung disease.

## References

[1] Luthra S, Rominski A, Sander P. 2018. The role of antibiotic-target-modifying and antibiotic-modifying enzymes in *Mycobacterium abscessus* drug resistance. Front Microbiol 9:2179. doi:10.3389/fmicb.2018.02179. 30258428 PMC6143652

[2] Daley CL, Iaccarino JM, Lange C, Cambau E, Wallace RJ, Andrejak C, Bottger EC, Brozek J, Griffith DE, Guglielmetti L, Huitt GA, Knight SL, Leitman P, Marras TK, Olivier KN, Santin M, Stout JE, Tortoli E, van Ingen J, Wagner D, Winthrop KL. 2020. Treatment of nontuberculous mycobacterial pulmonary disease: an official ATS/ERS/ESCMID/IDSA clinical practice guideline: executive summary. Clin Infect Dis 71:e1–e6. doi:10.1093/cid/ciaa241. 32628747 PMC7768748

[3] Wallace RJ, Jr, Meier A, Brown BA, Zhang Y, Sander P, Onyi GO, Bottger EC. 1996. Genetic basis for clarithromycin resistance among isolates of *Mycobacterium chelonae* and *Mycobacterium abscessus*. Antimicrob Agents Chemother 40:1676–1681. doi:10.1128/AAC.40.7.1676. 8807061 PMC163394

[4] Nash KA, Brown-Elliott BA, Wallace RJ, Jr. 2009. A novel gene, *erm*(41), confers inducible macrolide resistance to clinical isolates of *Mycobacterium abscessus* but is absent from *Mycobacterium chelonae*. Antimicrob Agents Chemother 53:1367–1376. doi:10.1128/AAC.01275-08. 19171799 PMC2663066

[5] Strnad L, Winthrop KL. 2018. Treatment of *Mycobacterium abscessus* complex. Semin Respir Crit Care Med 39:362–376. doi:10.1055/s-0038-1651494. 30071551

[6] Wu ML, Aziz DB, Dartois V, Dick T. 2018. NTM drug discovery: status, gaps and the way forward. Drug Discov Today 23:1502–1519. doi:10.1016/j.drudis.2018.04.001. 29635026 PMC6078814

[7] Baker SJ, Zhang YK, Akama T, Lau A, Zhou H, Hernandez V, Mao W, Alley MR, Sanders V, Plattner JJ. 2006. Discovery of a new boron-containing antifungal agent, 5-fluoro-1,3-dihydro-1-hydroxy-2,1-benzoxaborole (AN2690), for the potential treatment of onychomycosis. J Med Chem 49:4447–4450. doi:10.1021/jm0603724. 16854048

[8] Rock FL, Mao W, Yaremchuk A, Tukalo M, Crepin T, Zhou H, Zhang YK, Hernandez V, Akama T, Baker SJ, Plattner JJ, Shapiro L, Martinis SA, Benkovic SJ, Cusack S, Alley MR. 2007. An antifungal agent inhibits an aminoacyl-tRNA synthetase by trapping tRNA in the editing site. Science 316:1759–1761. doi:10.1126/science.1142189. 17588934

[9] Hernandez V, Crepin T, Palencia A, Cusack S, Akama T, Baker SJ, Bu W, Feng L, Freund YR, Liu L, Meewan M, Mohan M, Mao W, Rock FL, Sexton H, Sheoran A, Zhang Y, Zhang YK, Zhou Y, Nieman JA, Anugula MR, Keramane el M, Savariraj K, Reddy DS, Sharma R, Subedi R, Singh R, O'Leary A, Simon NL, De Marsh PL, Mushtaq S, Warner M, Livermore DM, Alley MR, Plattner JJ. 2013. Discovery of a novel class of boron-based antibacterials with activity against Gram-negative bacteria. Antimicrob Agents Chemother 57:1394–1403. doi:10.1128/AAC.02058-12. 23295920 PMC3591879

[10] Mendes RE, Alley MR, Sader HS, Biedenbach DJ, Jones RN. 2013. Potency and spectrum of activity of AN3365, a novel boron-containing protein synthesis inhibitor, tested against clinical isolates of *Enterobacteriaceae* and nonfermentative Gram-negative bacilli. Antimicrob Agents Chemother 57:2849–2857. doi:10.1128/AAC.00160-13. 23507283 PMC3716140

[11] Palencia A, Li X, Bu W, Choi W, Ding CZ, Easom EE, Feng L, Hernandez V, Houston P, Liu L, Meewan M, Mohan M, Rock FL, Sexton H, Zhang S, Zhou Y, Wan B, Wang Y, Franzblau SG, Woolhiser L, Gruppo V, Lenaerts AJ, O'Malley T, Parish T, Cooper CB, Waters MG, Ma Z, Ioerger TR, Sacchettini JC, Rullas J, Angulo-Barturen I, Perez-Herran E, Mendoza A, Barros D, Cusack S, Plattner JJ, Alley MR. 2016. Discovery of novel oral protein synthesis inhibitors of *Mycobacterium tuberculosis* that target leucyl-tRNA synthetase. Antimicrob Agents Chemother 60:6271–6280. doi:10.1128/AAC.01339-16. 27503647 PMC5038265

[12] Li X, Hernandez V, Rock FL, Choi W, Mak YSL, Mohan M, Mao W, Zhou Y, Easom EE, Plattner JJ, Zou W, Perez-Herran E, Giordano I, Mendoza-Losana A, Alemparte C, Rullas J, Angulo-Barturen I, Crouch S, Ortega F, Barros D, Alley MRK. 2017. Discovery of a potent and specific *M. tuberculosis* leucyl-tRNA synthetase inhibitor: (*S*)-3-(aminomethyl)-4-chloro-7–(2-hydroxyethoxy)benzo[c][1,2]oxaborol-1(3H)-ol (GSK656). J Med Chem 60:8011–8026. doi:10.1021/acs.jmedchem.7b00631. 28953378

[13] Tenero D, Derimanov G, Carlton A, Tonkyn J, Davies M, Cozens S, Gresham S, Gaudion A, Puri A, Muliaditan M, Rullas-Trincado J, Mendoza-Losana A, Skingsley A, Barros-Aguirre D. 2019. First-time-in-human study and prediction of early bactericidal activity for GSK3036656, a potent leucyl-tRNA synthetase inhibitor for tuberculosis treatment. Antimicrob Agents Chemother 63:e00240-19. doi:10.1128/AAC.00240-19. 31182528 PMC6658769

[14] Ganapathy US, Del Rio RG, Cacho-Izquierdo M, Ortega F, Lelievre J, Barros-Aguirre D, Lindman M, Dartois V, Gengenbacher M, Dick T. 2021. A leucyl-tRNA synthetase inhibitor with broad-spectrum anti-mycobacterial activity. Antimicrob Agents Chemother 65:e02420-20. doi:10.1128/AAC.02420-20. PMC8092876 33558292

[15] Yee M, Klinzing D, Wei JR, Gengenbacher M, Rubin EJ, Dick T. 2017. Draft genome sequence of *Mycobacterium abscessus* Bamboo. Genome Announc 5:e00388-17. doi:10.1128/genomeA.00388-17. 28522728 PMC5477336

[16] Aziz DB, Low JL, Wu ML, Gengenbacher M, Teo JWP, Dartois V, Dick T. 2017. Rifabutin is active against *Mycobacterium abscessus* complex. Antimicrob Agents Chemother 61:e00155-17. doi:10.1128/AAC.00155-17. 28396540 PMC5444174

[17] Dick T, Shin SJ, Koh WJ, Dartois V, Gengenbacher M. 2020. Rifabutin is active against *Mycobacterium abscessus* in mice. Antimicrob Agents Chemother 64:e01943-19. doi:10.1128/AAC.01943-19. 31767722 PMC6985736

[18] O'Dwyer K, Spivak AT, Ingraham K, Min S, Holmes DJ, Jakielaszek C, Rittenhouse S, Kwan AL, Livi GP, Sathe G, Thomas E, Van Horn S, Miller LA, Twynholm M, Tomayko J, Dalessandro M, Caltabiano M, Scangarella-Oman NE, Brown JR. 2015. Bacterial resistance to leucyl-tRNA synthetase inhibitor GSK2251052 develops during treatment of complicated urinary tract infections. Antimicrob Agents Chemother 59:289–298. doi:10.1128/AAC.03774-14. 25348524 PMC4291364

[19] Kim T, Hanh B-T-B, Heo B, Quang N, Park Y, Shin J, Jeon S, Park J-W, Samby K, Jang J. 2021. A screening of the MMV pandemic response box reveals epetraborole as a new potent inhibitor against *Mycobacterium abscessus*. Int J Mol Sci 22:5936. doi:10.3390/ijms22115936. 34073006 PMC8199016

